# Mucosal implications of oral Jak3-targeted drugs in COVID patients

**DOI:** 10.1186/s10020-025-01260-z

**Published:** 2025-05-23

**Authors:** Narendra Kumar, Daniel Segovia, Priyam Kumar, Hima Bindu Atti, Soaham Kumar, Jayshree Mishra

**Affiliations:** 1 ILR-College of Pharmacy, Texas A&M University Health Science Center, Kingsville, TX USA; 2 University of Pennsylvania, Philadelphia, PA USA; 3 Veterans Memorial High School, Corpus Christi, TX USA

**Keywords:** Keyword 1, Covid-19, Keyword 2, Jak3, Keyword 3, Mucosal -epithelial

## Abstract

The JAK family, particularly JAK3, plays a crucial role in immune signaling and inflammatory responses. Dysregulated JAK3 activation in SARS-CoV-2 infections has been associated with severe inflammation and respiratory complications, making JAK inhibitors a viable therapeutic option. However, their use raises concerns regarding immunosuppression, which could increase susceptibility to secondary infections. While long-term adverse effects are less of a concern in acute COVID-19 treatment, patient selection and monitoring remain critical. Furthermore, adverse effects associated with oral JAK3 inhibitors necessitate the exploration of alternative strategies to optimize therapeutic efficacy while minimizing risks. This review highlights the role of JAK3 in immune and epithelial cells, examines the adverse effects of oral JAK3 inhibitors in COVID-19 and other treatments, and discusses alternative therapeutic strategies for improving patient outcomes.

**Supplementary Information:**

The online version contains supplementary material available at 10.1186/s10020-025-01260-z.

## Introduction

### Current state of COVID virus

COVID-19 has been responsible for a worldwide epidemic since December 2019 and poses a worldwide medical threat. Individuals’ top respiration illnesses, fever, and serious pneumonia are caused by the extremely virulent encapsulated positive-strand RNA virus known as severe acute respiration syndrome coronavirus 2 (SARS-CoV-2). The virus uses host assets at every phase of its life cycle (Alam et al. 2021) and cellular kinase is necessary for their existence. In coronavirus diseases, these kinases are related to inflammatory processes, fibrosis, and signs like pneumonia and moreover spreading of the corona virus. Kinase inhibitors are, therefore, helpful in the fight against disease or epidemic since they have antibacterial, anti-inflammatory properties, anti-cytokine, and anti-fibrotic properties (Alam et al. 2021; Parvathaneni and Gupta 2020). Approximately between 25 and 30% of new drugs created by medical firms concentrate on protein kinases. Numerous potential kinase inhibitors helped the serious and sometimes fatal signs of COVID-19 (Bellera et al. 2021; Indari et al. 2021). Kinase inhibitors are used to reduce medical symptoms and immediately attack the infectious agent to avoid disease. Kinases are additionally employed to increase the effectiveness of specific SARS-CoV-2 therapies and investigations are still currently being conducted to determine the efficacy of the kinase inhibitors as an effective treatment (Chakraborty et al. 2021).

### COVID virus & tyrosine kinase signaling

#### Tyrosine kinases in viral entry and replication

AXL is a tyrosine kinase (TK) found on the cell membrane that regulates immune systems, development, division, and cell replication. Vitamin-K-reliant GAS-6 (amino acids development arrest-particular gene 6) is one ligand that activates AXL (Chakraborty et al. 2021). This simulates several subsequent signaling routes, like RAS/ERK, NF-B, p38, etc. AXL kinase was essential to COVID-19, allowing the virus to enter and influencing the antiviral activity of human Sertoli cells. SARS-CoV-2 uses AXL as a substitute receptor. By employing proteome techniques to analyze SARS-CoV-2 rise proteins that bind from cells lacking ACE2, it is possible to identify AXL. Three separate methods were used to determine the involvement of AXL in viral entry: adding AXL to AXL-negative HEK293 T cells, which allowed invasion; employing dissolved AXL to prevent virus; and breaking out AXL in ACE2-negative AXL-high cells, which prevented transmission. AXL transcription is significantly greater than ACE2 levels in respiratory and bronchi epithelium membranes. AXL's role in regulating SARS-CoV-2 disease is probably distinct from ACE2, as evidenced by the fact that it never shared with ACE2 or TMPRSS2 in the lungs or trachea, that downregulating AXL in ACE2-KO cells substantially lowered SARS-CoV-2 pseudo disease, and that never AXL nor ACE2 could prevent virus entry when the additional protein was overstated. Since SARS-CoV-2 binding, internalizing, and disease were significantly decreased but not eliminated in AXL-KO H1299 cells, there may be additional receptors besides AXL and ACE2 that control viral entry (Branco et al. 2020). Non-receptor TKs (NRTKs) are intracellular cytoplasm structures that are either nuclear-based or attached to the cell surface and transmit intracellular impulses. In cell signaling, NRTKs exhibit a wide range of functions. This involves controlling gene activity, for instance, by phosphorylating the membrane-based Janus kinase (JAK) with IL-6, which activates the signal transduction and activation of transcriptional (STAT) in COVID-19 (Shanker et al. 2024). The primary downward mechanism of type I IFN activity following viral replication involves the JAK-STAT signaling system, and a malfunction in this route was a key factor in the viral immunological evasion from routine immune monitoring. Few studies have examined the connection between COVID-19 and elevated ACE2 and JAK-STAT route transcripts. According to these findings, increased ACE2 levels may trigger the JAK-STAT signaling routes, which would alter the immunological system following an illness with SARS-Cov. More notably, the strong link between ACE2 & JAK-STAT signaling throughout the COVID-19 disease analysis was directly demonstrated by the above relationship involving ACE2 & IFN-JAK-STAT signaling being confirmed in a separate cell related to SARS-Cov and SARS-CoV-2 disease (Satarker et al. 2021).

#### Impact on immune response and cytokine storm

IL-6 has been shown to play an important role in the cytokine release syndrome (CRS) in the JAK-STAT signaling route, conferring a variety of biological activities. As previously stated, cytokines are high in COVID-19 patients, and these cytokines are recognized to perform an important part in the initiation of ARDS. This demonstrates that suppressing the cytokine storm through the JAK-STAT signaling route may be a potential strategy to enhance existing COVID-19 medical treatment options (Amini-Farsani et al. 2021). Several anti-inflammation cytokines are produced through the Bruton TK (BTK) route, and blocking BTK signaling lowers cytokines, which in turn has an anti-inflammation impact. It plays a part in mast cell activity through the high-affinity immunoglobulin E receptor. One important pathologic element of SARS-CoV-2 lung damage could involve defects in the control of the BTK signal in lung cells. Thus, blocking the BTK route may lessen the serious type of COVID-19's overactive and damaging immune response as well as the breathing issues that arise from it (McGee et al. 2021). The SARS-CoV-2 S and E proteins are ligands for TLR2, which binds to TLR1 and TLR6 to create heterodimers for signaling via TRIF and MyD88 (Zheng et al. 2021). To produce inflammation-related compounds, TLR2 regulates the stimulation of the NF-κB route. Two further investigations showed the binding location of SARS-CoV-2 S protein to TLR4, while computerized research indicated that SARS-CoV-2 S protein might bind effectively to TLR4. The viral S1 segment activates the NF-κB and mitogen-activated protein kinases (MAPK) routes, which have been linked to macrophage immunity stimulation.

#### Tyrosine Kinase Inhibitors (TKIs) as potential therapeutics

The possible use of baricitinib (JAK inhibitor) in the treatment of COVID-19 individuals (Spinelli et al. 2020; Cantini et al. 2020) conducted a pilot trial to examine the security and effectiveness of baricitinib treatment in 12 individuals with mild COVID-19. Individuals acquiring baricitinib showed higher clinical enhancements, with no infectious or hematologic complications noticed until 2 weeks afterward, resulting in medical professionals suggesting that short-term baricitinib application (1–2 weeks) is less probable to encourage important illness but could be capable of minimizing viral reproduction and the abnormal host inflammation action on medical dosage. Acalabrutinib (BTK inhibitor) was used in an observational trial by Roschewski, et al. (2020) on 19 inpatients who required extra oxygen because of serious COVID-19. After 10 to 14 days, most of the people who received treatment saw their inflammatory biomarkers revert to routine. Of 11 people who required additional oxygen, eight (72.7%) were able to breathe satisfactorily on room air after therapy. Out of eight individuals, 4 (50%) required ventilator-assisted exhumation. Lastly, after their medical care, twenty-five percent of intubated individuals inhaled freely in ambient air with no notable adverse outcomes. TKIs target protein kinases, although they typically disrupt several kinases (~ 10–100), increasing the potential for toxicity. Previous investigations found that approximately 5% of patients stopped using imatinib or multi-kinase inhibitors (MKIs) because of TKI-related side effects (Jacobs et al. 2021). Nevertheless, it is critical to recognize the strong link between therapeutic efficacy and the incidence of adverse responses. The adverse reaction characteristic is commonly employed as a tracking instrument to determine the intended results of cancer treatment. Nevertheless, a positive association was found between the percentage of kinases hindered and the level of their hazardous capacity.

### COVID virus & non-tyrosine signaling

#### Serine/threonine kinase pathways in SARS-CoV-2 infection

A vital cell signaling route, the PI3 K/Akt/mTOR route controls several cell functions, including anabolism, nutrition intake, cell development, distinction, existence, proliferating, and movement. This has contributed to the theory that SARS-CoV-2 disease could be improved by either one or two inhibitors of this route. The authorized use of PI3 K, Akt, and mTOR individual inhibitors is for the therapy of various illnesses, mostly but not solely neoplasm. While some combo inhibitors are in clinical testing, just a limited number have received approval for medical usage (Basile et al. 2022). Numerous cellular functions depend on intracellular signaling routes that are controlled by MAPKs. The MAPK class in animals primarily consists of p38 MAPKs, c-Jun N-terminal kinases, and external signal-controlled kinases. Certain external stimuli trigger the MAPK pathway action, which involves the successive activity of MAPK kinase and MAPK kinase (MAPKKK), which in turn triggers the release of certain MAPK. In addition to SARS-CoV disease, the p38 MAPK route was linked to inflammation damage in COVID-19 sufferers'hearts and lungs (Roy et al. 2021).

#### AMPK and other kinases in host response

An intracellular serine/threonine kinase called AMPK plays a crucial role in regulating metabolism and preserving cellular power. By altering cellular metabolism to produce power via catabolic routes like glucose absorption and by inhibiting unnecessary anabolic activities like protein, lipid, and carbohydrate production, AMPK is triggered by metabolism strain and works to maintain power equilibrium (Huang et al. 2022). The PKA and PKC routes are two of the signaling mechanisms that control the synthesis of IL-6. PKC inhibitors decrease SARS-CoV-2 growth in BHK-21 cells. A SARS-CoV-2 replica, which contains virus NS proteins linked through a nano luciferase, was employed to replace the architectural proteins and several auxiliary proteins to investigate if PKC inhibitors impact SARS-CoV-2 replicating. PKC inhibitors work at the starting point of the replicating of SARS-CoV-2. The kinetic luciferase activity of the replication in BHK-21 cells was observed to ascertain the stage at which the PKC inhibition works (Huang et al. 2022).

### Crosstalk between tyrosine and non-tyrosine kinase pathways during covid infection

The JAK/STAT and PI3 K/AKT/mTOR routes are the primary signaling processes for numerous development variables and cytokines, and their activation promotes cell proliferation, distinction, movement, and death. Nevertheless, numerous malignancies, such as leukemia, have continuous activation of the JAK/STAT and PI3 K/AKT/mTOR routes because of inherited occurrences like cytokine receptor alterations and abnormal chromosomal transfers. C-Myc was overproduced by continuous activity of the JAK/STAT and PI3 K/AKT signaling routes (Mahjoor et al. 2023). As a dual-targeting treatment for COVID-19, reseparate reduces virus transmission and inhibits macrophage-mediated proinflammatory immunity (Mishra et al. 2017). Rone-parstat's secure patient characteristics and dual-targeting impact on virus disease and macrophage-triggered inflammation cytokine discharge may be helpful in this situation. To sum up, the preliminary evidence indicates that heparinase is a focus for the pathophysiology of SARS-CoV-2 and that Reseparate may be a potential dual-targeting drug for COVID-19. Given the enormous role of mucosal Jak3 in inflammation, the present article highlights the importance of Jak signaling during different stages of COVID-19 infections and the essential roles of mucosal epithelial Jak3 which are anatomically juxtaposed to the immune cell Jak3, in COVID pathogenesis (Kumar et al. 2021). Jak inhibitors are mostly oral (Li et al. 2022) and encounter lung and/or intestinal mucosal epithelial cells first before targeting immune cell Jak3 inhibition and taming the cytokine storm. Our group has extensively worked on mucosal epithelial Jak3 including its role in intestinal restitution and redifferentiation of mucosal epithelial cells following restitution not to mention its role in epithelial mesenchymal transition, cell homeostasis, and barrier function restoration (Kumar et al. 2021; Kumar et al. 2007; Kumar et al. 2022; Mishra et al. 2019; Mishra et al. 2017; Mishra et al. 2013). As a part of the scientific community, it is important to discuss and understand the functional consideration for mucosal epithelial-specific Jak3 and their consequences in designing and developing the Jak3 or JAK-STAT signaling targeted drugs for reducing cytokine storm to combat covid. Based on published literature and current understanding on the emerging roles of mucosal epithelial Jak3 functions, additional preliminary data are provided on constant communication and crosstalk between mucosal epithelial Jak3 and immune cell Jak3 in covid pathogenesis that can shed new lights on Jak3-mediated interventions. Additionally, the implications of Jak3-domain structures other than those of the kinase domain are also discussed in context of targeted interventions for the emerging variants of COVID through rational drug designing.

### Covid drugs targeting cytokine cascade

A growing body of clinical data suggests that a cytokine storm is an excessive immune response associated with COVID-19 severity and the main cause of death from COVID-19 (Ye et al. 2020). A key player in this process is interleukin-6 (IL-6), a pro-inflammatory cytokine and targeting this key player and treatment of the cytokine storm are important components for rescuing patients with severe COVID-19. In COVID-19, elevated levels of IL-6 contribute to the hyperinflammatory state observed in cytokine storms. At the molecular level, IL-6 binds to its receptor (IL-6R), forming a complex that interacts with the signal transducer gp130, leading to the activation of inflammatory pathways (Li et al. 2023). Evidence suggests that the hyperinflammatory response secondary to SARS-CoV-2 infection, are responsible for multi-organ damage in patients with COVID-19 (Li et al. 2023). For these reason, numerous randomized clinical trials are currently underway to explore the effectiveness of biopharmaceutical drugs, such as, interleukin-6 inhibitors, interleukin-1 blockers, Janus kinase inhibitors, in COVID-19. Tocilizumab is a humanized monoclonal antibody that targets the IL-6 receptor (IL-6R), thereby inhibiting IL-6-mediated signaling (Ali et al. 2020). Tocilizumab blocks pro-inflammatory activity of interleukin-6 (IL-6), involved in pathogenesis of pneumonia the most frequent cause of death in COVID-19 patients. Although with limitations of a single-arm study, performed in an extremely challenging time and environment, use of tocilizumab, even when corticosteroids are used, while waiting for publication of phase 3 results. Administration of tocilizumab has been linked to a decrease in mortality rates among patients exhibiting high levels of IL-6 and requiring oxygen support. The effectiveness of tocilizumab may depend on the timing of its administration, with earlier intervention during the hyperinflammatory phase potentially yielding better outcomes (Piccirillo et al. 2020; Leaf et al. 2021; Chiodini et al. 2020). Canakinumab, a human monoclonal antibody targeting interleukin-1 beta was used to improve respiratory function and laboratory parameters compared with standard therapy (hydroxycloroquine plus lopinavir/ritonavir) (Leaf et al. 2021; Ucciferri, et al. 2020; Falasca et al. 2021). In hospitalized adult patients with mild or severe non-ICU COVID-19, canakinumab is a valid therapeutic option as Canakinumab therapy caused rapid and long-lasting improvement in oxygenation levels in the absence of any severe adverse events. Finally, Janus kinase (JAK) inhibitors, which may have a role in blocking the fibrotic evolution in some patients, are being evaluated (Mahjoor et al. 2023). The JAK/signal transducers and activators of transcription STAT-pathway mediates the signaling of multiple cytokines, therefore interrupting this pathway may be an attractive strategy to modulate the immunopathology observed in SARS-CoV-2 infection (Satarker et al. 2021). Besides, many JAK inhibitors exhibit antiviral effects when administered at therapeutic doses, by targeting host factors that viruses use for cell entry (Jain et al. 2023). Baricitinib is an oral JAK inhibitor, that inhibits the JAK signal transducer and activator of transcription pathway which are under investigation in some clinical trials on COVID-19 patients. A pilot study from Italy showed a significantly improvement in clinical and laboratory parameters in patients treated with baricinib (Singh, et al. 2021) Ruxolitinib, another JAK-kinase inhibitor, showed in COVID19 patients with hyperinflammation, to be safe and prevent multiorgan failure (Han et al. 2022; Weinstein et al. 2025; Ucciferri et al. 2020).

### Genetic variations in JAK and COVID-19 severity and susceptibility

Genetic variations in human host genes play a significant role in susceptibility, severity, and outcomes of, infectious diseases—including COVID-19. Among the most critical pathways involved in host immune response is the Janus kinase (JAK)/signal transducer and activator of transcription (STAT) signaling cascade. This evolutionarily conserved pathway is activated by a range of cytokines, interferons, and growth factors, serving as a central mediator of immune regulation (Rao et al. 2023). Functionally, relevant mutations and polymorphisms in JAK family proteins, including TYK2, JAK1, JAK2, and JAK3, have been associated with diverse immune-related diseases and have now been implicated in COVID-19 severity and susceptibility (Kosmicki et al. 2024; Singh et al. 2021). The clinical relevance of the pathway is underscored by the development of a new class of targeted therapeutics—JAK inhibitors—currently in use for inflammatory diseases and under investigation for COVID-19 treatment. Recent studies have identified specific variants of Tyk2 associated with COVID-19 outcomes. TYK2 p.Gly363Ser and STAT2 p.Met594Ile have been linked to increased susceptibility to SARS-CoV-2 infection (Benmansour et al. 2024; Jiang et al. 2023; Castro et al. 2025). Genome-wide association studies (GWAS), including data from the COVID-19 Host Genetics Initiative, have demonstrated that loss-of-function mutations in TYK2 are protective against severity disease. TYK2 is critical in type I interferon (IFN) and IL-12 signaling; thus, its inhibition may reduce hyperinflammatory responses such as cytokine storms which positions TYK2 inhibitor**s** as promising dual-purpose therapeutics—attenuating inflammation while minimizing the risk of immune suppression (Guo et al. 2022). Further bioinformatic analyses have highlighted the potential role of rare variants in interferon receptor genes IFNAR1 p.Val168Leu, is associated with a reduced risk of severe COVID-19, while rare pathogenic variants in both IFNAR1 and IFNAR2 may impair antiviral signaling. Other JAK family members also play essential roles in immune regulation. While JAK1 and JAK3 have been less prominently implicated in GWAS, their roles remain biologically significant. JAK1 is indispensable for type I IFN signaling, and functional defects could impair viral clearance (Hasselbalch et al. 2021; Hammersen et al. 2023). Gain-of-function mutations like JAK2 V617 F result in constitutive activation of the pathway and may exacerbate hyperinflammatory responses, thereby worsening COVID-19 pathology (Elbadry et al. 2022; Pelkey et al. 2022). JAK3, primarily expressed in immune cells, is associated with immunodeficiency syndromes, which may influence COVID-19 outcomes depending on the inflammatory context. Similarly, mutations in MPL associated with Myeloproliferative neoplasms (MPNs) (e.g., W515L/K/A) and deletions in LNK, a negative regulator of JAK-STAT signaling, contribute to aberrant pathway activation, with potential consequences for disease severity (Lucijanic et al. 2021; Harrington et al. 2021). So, host genetic variation in the JAK-STAT signaling axis—particularly in TYK2, IFNAR1/2, and JAK2—profoundly influences COVID-19 susceptibility and severity that lays the groundwork for not only personalized therapeutic strategies, including early immunomodulation tailored to patients with pro-inflammatory JAK variants but also for impaired antiviral signaling. TYK2 polymorphisms have been linked with autoimmune diseases such as systemic lupus erythematosus (SLE), psoriasis, and multiple sclerosis (MS). A well-studied TYK2 SNP, rs34536443 (P1104 A), is a hypomorphic allele, which decreases IL-12 and IL-23 signaling, conferring protection against autoimmunity but susceptibility to infection (Dendrou, et al. 2016). STAT3 gain-of-function (GOF) mutations were found to occur in early-onset autoimmunity, lymphoproliferation, and enteropathy. The mutations result in hyperactivation of STAT3-dependent genes, which contributes to immune dysregulation syndromes (Zhou et al. 2016). STAT1 GOF mutations lead to interference with STAT1 dephosphorylation, leading to enhanced interferon signaling manifests, chronic mucocutaneous candidiasis and autoimmunity. Alternatively, STAT1 loss-of-function leads to enhanced susceptibility to mycobacterial and viral infections (Zhang et al. 2008).

### Immune vs mucosal cell Jak3

#### Immune cell Jak3

Is a non-receptor tyrosine kinase of the Janus family, is involved in cell growth, survival, development, and differentiation of a variety of cells but are critically important for immune cells and hematopoietic cells. Deficiency of Jak3 results in defined clinical disorders, particularly, inactivating Jak3 mutations leads to severe combined immunodeficiency syndrome and has been viewed as an excellent therapeutic target for the development of a new class of immunosuppressive drugs. In fact, several companies are developing JAK3 inhibitors, and Phase II studies are underway. Tofacitinib, as the first JAK inhibitor, is approved for rheumatoid arthritis therapy. Also, many other JAK inhibitors have been proven or are in various phases of clinical trials for various diseases. At present, small-molecule JAK inhibitors are considered as a novel category of drugs in the treatment of cancer and auto immune diseases.

#### Mucosal functions of Jak3

Jak3 is expressed in non-hematopoietic cells, where it plays crucial roles in mucosal wound repair, cell homeostasis, efflux function, and barrier integrity (Kumar et al. 2021; Kumar et al. 2007; Mishra et al. 2012; Shao et al. 2023; Momen et al. 2024). It contributes to mucosal wound healing by reorganizing actin, facilitating epithelial motility through filopodial and lamellipodial extensions in enterocytes (Kumar et al. 2007). Additionally, Jak3 enhances IL-2-induced villin phosphorylation, promoting gastrointestinal cell wound healing. Intestinal mucosal homeostasis is maintained through a delicate balance of crypt cell proliferation, migration along the crypt-villus axis, differentiation, and eventual apoptotic shedding at the villus tip. IL-2 activates Jak3, which regulates mucosal homeostasis through post-translational and transcriptional mechanisms, including its interaction with the adapter protein p52ShcA (Mishra and Kumar 2014). Jak3 is also critical for barrier function, adherent’s junction (AJ) formation, epithelial-mesenchymal transition (EMT), and colonic polyp development by interacting with and phosphorylating β-catenin (Mishra et al. 2017). Furthermore, Jak3 influences intestinal drug efflux and barrier integrity through its interaction with and phosphorylation of the efflux protein BCRP (Mishra et al. 2019). This interaction enhances BCRP’s association with membrane-localized β-catenin, which is essential for BCRP expression, surface localization, and efflux functionality.

## COVID-19 and Jak signaling

Reported first in late 2019 in China, COVID-19 is a respiratory infection caused by SARS-CoV-2, (Zhou, et al. 2020) The initial symptoms include cold, dry cough followed by sputum, shortness of breath, and dyspnea. However, upon respiratory failure the disease worsening leading to acute respiratory distress syndrome (ARDS) and cytokine storm causing hyperinflammation and even death (Zhang et al. 2022) The cytokine storm is characterized by increase in pro-inflammatory cytokine including IL-1, IL-2, IL-6, IL-7, IL-8, IL-10, TNF-α, GCS-F, INF-γ inducible protein 10, MCP-1, and MIP-1α (Mehta et al. 2020) The cytokine storm is one of the key event implicated in several of the COVID-19 complications including lung injury, ARDS, acute kidney and cardiac injury leading to multi-organ failure eventually leading to death in COVID-19 patients (Mehta et al. 2020; Yang et al. 2020) Among the cytokines, the key prognosis markers are elevated serum levels of IL-6 and IFN-gamma. The high mortality rate has been attributed to cytokine storm led hyperinflammation vascular disorders culminating in pulmonary embolism, cerebral thromboembolism and multisystem failures (Tanaka 2023). Since Jaks play a pivotal role in majority of cytokine signaling through JAK-STAT pathways in COVID-19 pathophysiology, an understanding of its beneficial and detrimental impacts is warranted.

### Overview of SARS-CoV-2 and Jak-targeted drugs

The World Health Organization continues to classify COVID-19 as a worldwide pandemic, still with positive cases in the developed and developing countries (Cucinotta and Vanelli 2020; Solis Arce et al. 2021; Smith et al. 2020). Since the beginning of the pandemic, multiple vaccines have been released to prevent COVID-19 (Andrews, et al. 2022; Chung et al. 2021; Lopez Bernal, et al. 2021; Thompson et al. 2021; Hall et al. 2022) and many antiviral medications have been developed to reduce the progression of COVID-19 infection, as suggested by clinical and randomized controlled trials (Forchette et al. 2021; Kumari et al. 2022; Butler et al. 2023; Beigel et al. 2020; Rosas et al. 2021; Singh et al. 2022; Cao et al. 2023). Though several resources are available to the public, the number of cases is still significant due to the many variants that have appeared including Delta and Omicron (Andrews, et al. 2022; Lopez Bernal, et al. 2021; Forchette et al. 2021; Pilz et al. 2022). Since the end of COVID 19 complications is nowhere in sight it is important to delineate and comprehend the adverse health implications and develop better interventions to treat these diseases. Cytokine storm is one of the various hallmarks for COVID related death (Luo et al. 2020; Que et al. 2022; Ragab et al. 2020; Rincon-Arevalo et al. 2022; Geng et al. 2021; Karki, et al. 2021). Prior to COVID, Jak inhibitors were approved by the FDA for autoimmune conditions such as rheumatoid and psoriatic arthritis (Berteloot, et al. 2021; Chang and Lai 2019; Sanachai et al. 2020; Liang et al. 2020; Quero et al. 2019; Long et al. 2022; Dai, et al. 2021). Reports suggest the potential of Jak inhibitors and particularly Jak3 inhibitors, in targeting the immune cell thereby reducing the cytokine storm and ameliorating some of the COVID related symptoms (Luo et al. 2020; Que et al. 2022; Levy, et al. 2022; Kramer, et al. 2022; Sparks et al. 2021; Kalil et al. 2021; Marconi et al. 2021; Murugesan et al. 2022; Sonkar et al. 2021; Sarmiento et al. 2021; Guimaraes et al. 2021; Maslennikov et al. 2021). However, Jak3 is also present in other cell-types, importantly in the intestinal mucosal epithelial cells and in the nasopharyngeal mucosal surface which is an important entry point for COVIDs (Kumar et al. 2021). Reports suggest COVID-19 impacts Jak3 signaling and its inhibition in the immune cells may have potential beneficial effects (Rincon-Arevalo et al. 2022; Geng et al. 2021; Karki, et al. 2021; Li et al. 2021; Xia et al. 2020; Zhang, et al. 2022; Tran et al. 2021; Feng et al. 2022). However, information on the role of mucosal Jak3 in COVID prevention and/or treatment is lacking.

### Jak signaling and the breach of primary barrier during COVID-19

In general, almost all viral infections including COVID-19 have 4 stages that involve viral- entry, replication, assembly, and release. COVID-19 infections require breach of the very first barrier which is the mucosal epithelial cells of both respiratory and gastrointestinal systems. SARS-CoV-2 entry involves attachment of the viruses through spike 1 protein to the alveolar mucosal epithelial cells expressed angiotensin-converting enzyme 2 (ACE2) receptors. The alveolar epithelium secretes mucus, which traps inhaled particles and bacteria and prevents them from reaching the lungs. ACE-2 surface expression is also responsible for viral entry into a variety of other cell types including renal epithelial cells, endothelial cells, cardiac myocytes, and immune cells such as macrophages and monocytes in the spleen and lymph nodes. viral entry to any of these cell types requires breach of the very first barrier which is the mucosal epithelial cells of both respiratory and gastrointestinal systems. The role of JAK signaling in the transcription and activation of ACE2 in lung epithelium has also been reported (Lee et al. 2020). Reports also suggest (Luo et al. 2021) a close correlation between ACE2 and JAK-STAT pathway in the regulation of immune responses and a role of overexpressed ACE2 in downstream JAK-STAT signaling to modulate SARS-CoV-2 induced inflammatory responses. Recently, (Al-Ani et al. 2022) reported the involvement of lung angiotensin II to type 1 receptor (AT1R)-JAK-STAT axis in lipopolysaccharides (LPS)-induced cytokine storm and resulting acute lung injury and coagulopathy corresponding with moderate-to-severe COVID-19 in humans. Similarly, Interferon-alpha 2 is reported to further augment the viral load by upregulating the ACE2 expression in a loop-back mechanism in different cell systems (Wilk et al. 2020).

### Jak signaling in COVID-19 escaping natural immunity and viral replication

Jak signaling plays a protective role during viral infection which is indicated by the fact that during the early stages of infection, SARS-CoV-2 induces negative regulator proteins SOCS3 to inhibit JAK2-STAT3 signaling to escape antiviral responses and replicate freely (Zizzo et al. 2022). In addition, SARS-CoV-2 infection is characterized by the reduced immune cell counts including NK cells, B cells, CD4^+^ and CD8^+^ T cells (Zheng et al. 2022) which are not only found in human with Jak3 mutations but also in Jak-KO mice models. Moreover, SARS-CoV-2 virus infected patients show impaired type I interferons mediated antiviral response which are in turn mediated by JAK-STAT pathway (Walz et al. 2021).

### Jak signaling in cytokine storm and ARDS symptoms of COVID-19

**Fig. 1:**
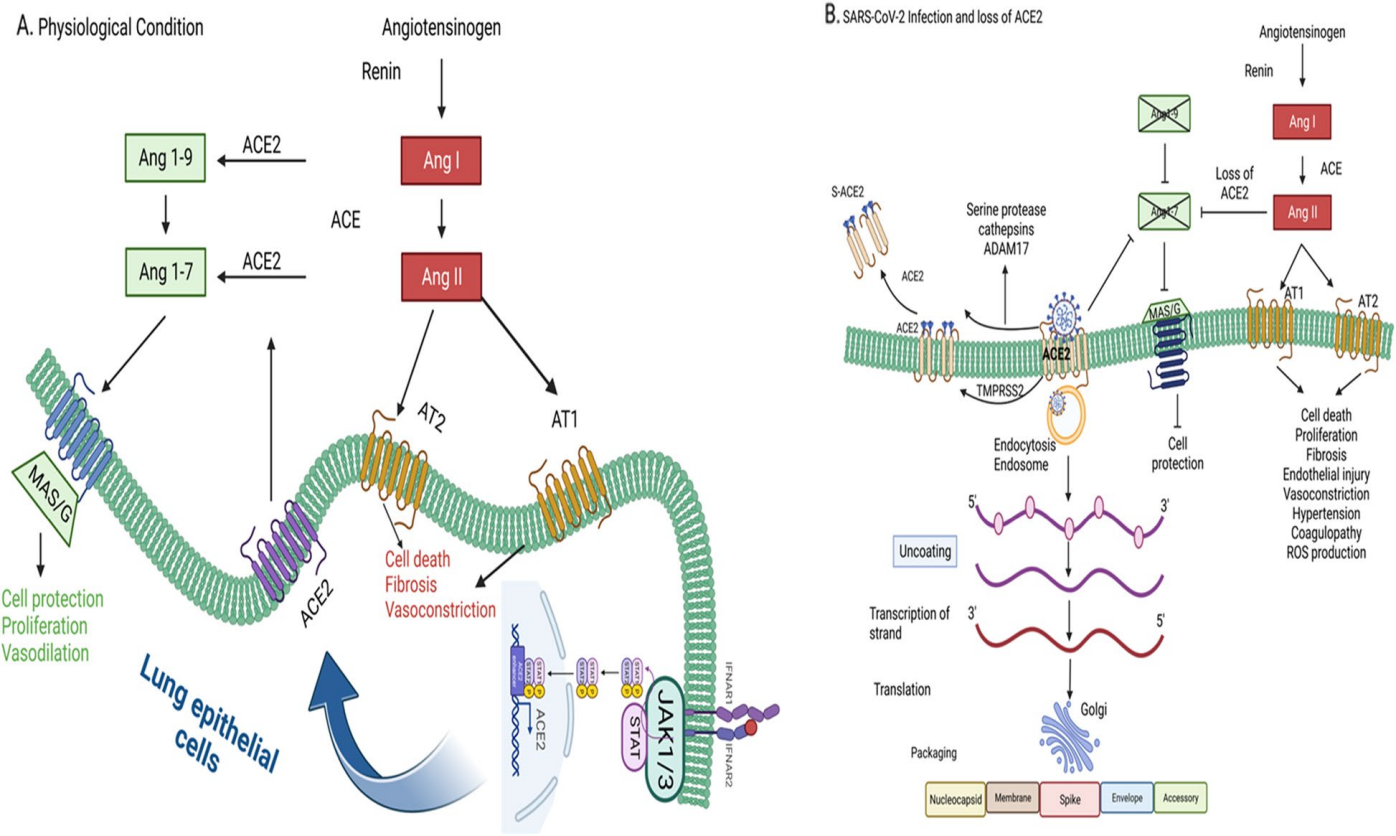
Model for Jak3-mediated normal lung physiology and its implications during COVID-19 infections mediated compromised lung functions. **A** Under normal physiological conditions, Jak-3 activation facilitated the surface expression of ACE2 that maintains lung epithelial homeostasis and vasodilation-mediated normal lung functions through Ang 1–7 formation and its interactions with its receptor MAS/G. **B** SARS COVID-19 infection through its entry into lung mucosal epithelial lining through ACE2 however leads to loss of ACE2, accumulation of Ang-II, activation of AT1 and AT2 by Ang-II, and mucosal epithelial cell death, fibrosis, vasoconstriction, hypertension, and coagulopathy-associated health complications

After viral entry through mucosal breach followed by escaping the natural immunity and viral replications, COVID-19 patients show broad spectrum of clinical symptoms that emanates from significant increase in pro-inflammatory cytokines and chemokines clinically termed as cytokine storm. Among the primary and life threatening symptoms include acute respiratory distress syndrome (ARDS) and multi-organ failure and if uncontrolled, leads to subsequent death (Rarani et al. 2022). Among the elevated cytokines (IL-2, IL-4, IL-6, IL-7, IL-10, TNF-alpha, IFN-gamma) and chemokines (CCL2, CCL8), see during cytokine storm (Khaledi et al. 2022) the same JAKs which were initially involved in reinforcing mucosal barrier and natural immunity becomes detrimental and play a key role in immunopathology of COVID-19 disease through increased cytokine production via IL-6/GM-CSF/JAK-STAT axis. In addition, these Jak-signaling also facilitate recruitment of macrophages, monocytes, neutrophils, natural killer cells, lymphocytes, and dendritic cells augmenting cytokine production leading to ARDS and even death. Among the examples of individual Jaks involved include JAK1,2 mediated localized complement hyperactivation (Yan, et al. 2021). IL-2 induced JAK1,3 mediated cytotoxicity of NK cells, and IL-6 induced JAK1 mediated IL-15 production and NK cell development, growth, and functioning (Gotthardt et al. 2019). Another key growth factor TGFβ that stimulate pulmonary fibrosis during COVID-19 acute lung injury are produced due to JAK1-STAT1 dysregulation led hyperactivation of STAT3 which is a key transcription factor for the overexpression of harmful IL-6 that stimulates transforming growth factor-1beta (Matsuyama et al. 2020) (Fig. 1).

### Current state of Jak inhibitor drugs

**Fig. 2:**
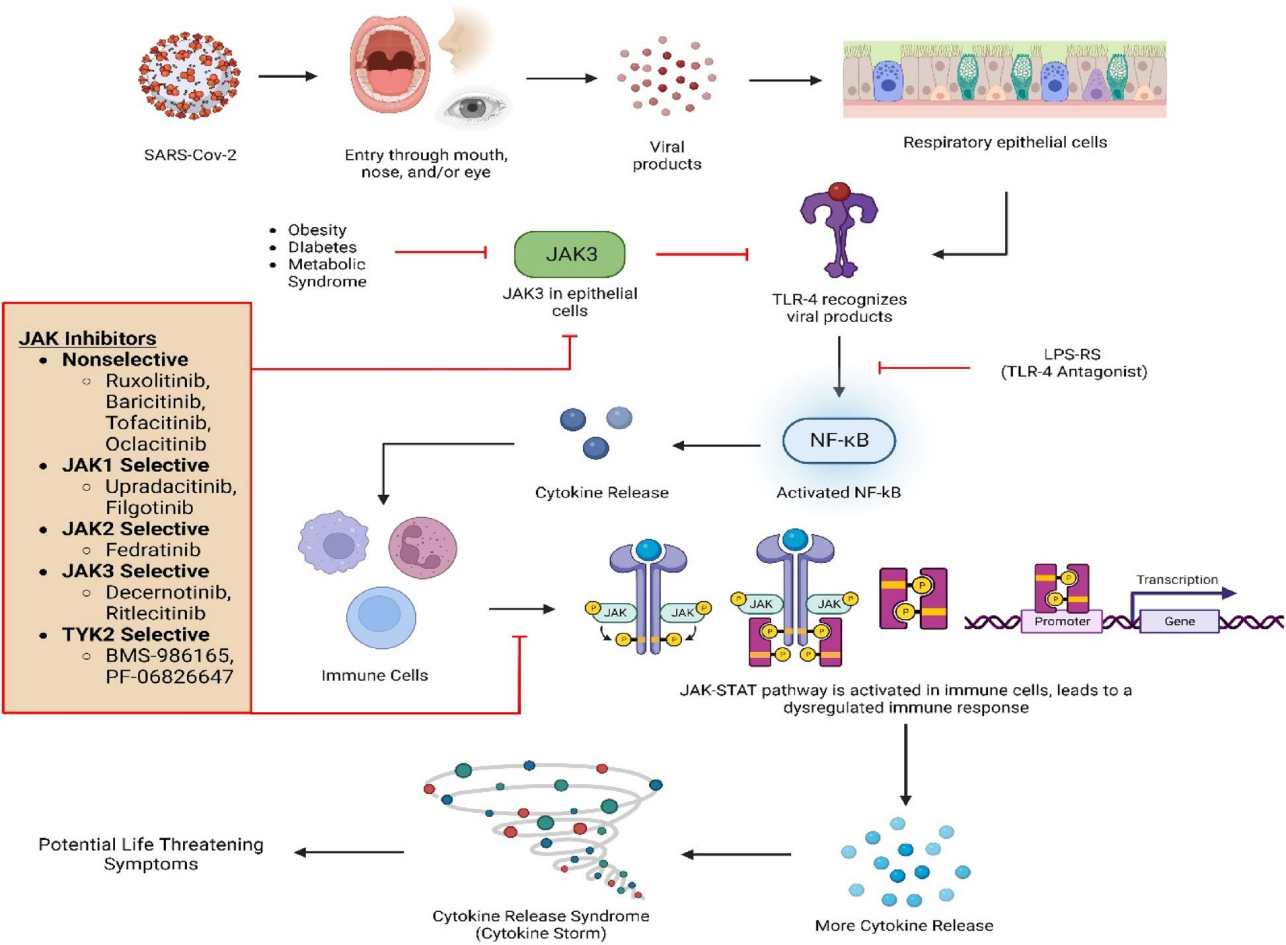
Jak3 and Covid in Epithelial and immune cells. Model for the central roles of Jak3 in respiratory epithelial cells (REC) and immune cells and its implication during SARS COVID-19 infection. While in REC, Jak3 suppresses COVID-mediated TLR activation and reduced cytokine production, and these are compromised due to reduced Jak3 expression led increased cytokine production through NF-kB in patients with obesity, diabetes, and metabolic syndrome. These cytokines in turn further activate the circulating immune cells causing cascade effects of cytokine syndrome. Therefore, Jak3 targeted drugs have limited efficacies due to suppression of Jaks in both REC and immune cells where though it impacts the cytokine syndrome, however, compromises lung functions due to loss of protective effects by REC

Though the crystal structure of the kinase domain for all JAKs has been determined in the active state conformation, the three-dimensional structure of full length JAKs is still unclear. For that reason, currently available all Jak inhibitors (Jakinibs) are screened against only the truncated kinase domain while human body expresses full length Jaks where other domains of Jaks influence the binding of not only Jak substrates but also its own activation through binding of ATP. Interestingly, a patent exists for screening Jak3 inhibitors using full-length Jak3 (US patent# US 9,739,779). Moreover, the Jak inhibitors are usually categorized as first- or second- generation Jakinibs. The first-generation Jakinibs works through competitive inhibition of ATP binding site on the JH1 kinase domain. Since the ATP-binding site is highly conserved among JAKs, the first-generation Jakinibs targeted multiple Jak member both in vitro and in vivo (Ferrao and Lupardus 2017). These resulted in the inhibition of multiples pathways regulated by different Jaks in several tissues resulting in off-target binding of the currently available drugs/inhibitors and consequent adverse effects (Clark et al. 2014). Subsequent efforts were made to develop next-generation Jakinibs to increase the specificity and selectivity to JAKs which were still targeted to the ATP-binding site through non-covalent competitively and non-covalent binding though some also targeted the JH2 pseudo-kinase domain of JAK (Deucravacitinib) while other inhibited the same truncated domain of JAKs through covalent binding (Ritlecitinib) (Casimiro-Garcia et al. 2018; Burke, et al. 2019). Since the binding site and the mode of screening largely remains unchanged and conserved across several Jaks, it is unlikely that the specificity and the adverse events would improve significantly (Fig. 2).

### Structure of Jak1, jak2, Jak3 and Tyk2 proteins and their interactions with specific inhibitors

Understanding the structural domains of Jaks and their roles in its activation and inhibition is crucial for the rational design of selective inhibitors. The kinase domain of JAK1 contains an ATP-binding site that is a primary target for small-molecule inhibitors designed to modulate JAK1 activity. High-resolution crystal structures have revealed that inhibitors bind deep within this ATP-binding pocket, interacting with key residues such as Glu957 and Leu959 in the hinge region. These interactions are specific and crucial for the potency and selectivity of the inhibitors (Raivola et al. 2019). By targeting specific residues within the ATP-binding site, it is possible to develop compounds that effectively modulate JAK1 activity with reduced off-target effects, enhancing therapeutic efficacy in treating diseases involving dysregulated JAK-STAT signaling.​ Baricitinib (JAK1/2) and ruxolitinib (JAK1/2) have been used in severe COVID-19 to dampen hyperinflammation (Hasselbalch et al. 2021; Vannucchi, et al. 2021). pure JAK1 inhibitors like Upadacitinib haven’t seen widespread COVID-19 use, the therapeutic potential of this drug against Covid needs to be investigated (Sonkar et al. 2021).

The kinase domain of JAK2 contains an ATP-binding site that is the primary target for small-molecule inhibitors designed to modulate JAK2 activity. These inhibitors can be categorized based on their binding modes.​ Type I Inhibitors bind to the active conformation of the kinase domain, occupying the ATP-binding site (Ruxolitinib and fedratinib) which are used clinically to treat myeloproliferative disorders (Vannucchi, et al. 2021; Tefferi et al. 2023; Keenan et al. 2024; Naik et al. 2022). Type II Inhibitors target the inactive conformation (DFG-out) of the kinase domain, binding to the ATP-binding site and an adjacent hydrophobic pocket.​ (NVP-BBT594 and NVP-CHZ868) which exhibit selective inhibition of JAK2 (Keenan et al. 2024). Additionally, allosteric inhibitors that bind to sites remote from the active site are being explored to achieve greater specificity and reduce off-target effects. These inhibitors offer a potential advantage by modulating JAK2 activity without directly competing with ATP binding (Liang et al. 2023).

The structural organization of TYK2 provides multiple targets for small-molecule inhibitors such as ATP-Competitive Inhibitors bind to the ATP-binding site within the kinase domain (JH1), directly competing with ATP and inhibiting kinase activity (Ropsacitinib and Brepocitinib) which target the catalytic ATP-binding sites, have shown efficacy in modulating TYK2 activity (Kimball, et al. 2024; Zhou et al. 2022). Allosteric Inhibitors bind to the pseudokinase domain (JH2), inducing conformational changes that stabilize the autoinhibited state of TYK2, thereby preventing activation.​ Targeting the pseudokinase domain offers a novel approach to achieve specificity, as demonstrated by the development of allosteric inhibitors like deucravacitini. Deucravacitinib is a selective TYK2 inhibitor that binds allosterically to the JH2 domain, preventing receptor-mediated activation. ​ These inhibitors provide therapeutic potential with improved selectivity and reduced off-target effects compared to traditional ATP-competitive inhibitors (Lebwohl et al. 2024). In summary, the detailed structural organization of TYK2, particularly its pseudokinase and kinase domains, provides a foundation for developing targeted therapies aimed at modulating TYK2-mediated signaling pathway.

### Implications for drug resistance and therapeutic innovation

Structural changes, e.g., point mutations arising within the kinase or pseudo kinase domains, may confer resistance to JAK inhibitors. For example, mutations JAK1 Y1034 C or JAK3 L857Q prevent drug binding but retain kinase activity (Koppikar et al. 2012). Comprehension of the three-dimensional structure and conformational dynamics of JAK domains is therefore necessary for the control of resistance mechanisms and next-generation inhibitor design. Advances in cryo-electron microscopy and X-ray crystallography have illuminated the full-length architecture of JAKs in complex with receptors and inhibitors, illuminating inter-domain communication and allosteric sites accessible to therapeutic intervention (Glassman et al. 2022). Resistance to JAK inhibitors can be caused by point mutations in the pseudo kinase domain or ATP-binding site. These mutations can disrupt drug binding but preserve kinase activity. For instance, mutations such as JAK1 Y1034 C and JAK2 R683S have been shown to decrease sensitivity to inhibitors (Downes et al. 2021).

### Therapeutic combinations of JAK inhibitors and other Covid drug

Monotherapy is not always the best option, however, and combination therapy regimens with other anti-inflammatory drugs are used to optimize clinical responses or reduce corticosteroid therapy. The therapeutic basis for combination of JAK inhibitors with drugs like corticosteroids, methotrexate (MTX), or biologics is their complementary mechanism of action and synergistic immunosuppressive potential. To improve the clinical outcomes such as oxygenation and survival in moderate to severe cases of Covid, Corticosteroids (like dexamethasone) is used in combination with JAK inhibitors to reduce systemic inflammation associated with the cytokine storm (Meyer et al. 2020). However, there is a risk for additive immunosuppression and secondary infections. Corticosteroids have broad anti-inflammatory actions through the inhibition of pro-inflammatory gene transcription and inhibition of varied immune cell functions. Combination with JAK inhibitors, which block cytokine receptor-mediated JAK-STAT signaling, can result in synergistic inhibition of inflammatory mediators. For instance, combination therapy with baricitinib and dexamethasone in COVID-19-associated cytokine storm led to a significant reduction in mortality. The COV-BARRIER trial demonstrated that the addition of baricitinib to standard of care (including corticosteroids) reduced 28-day mortality by 38.2% compared to placebo (Marconi et al. 2021). Compared to Remdesivir alone, Baricitinib and Remdesivir along with corticosteroids showed quicker recovery and less progression to ventilation in the COVID-19 (ACTT-2 Trial) (So et al. 2021). Subgroup studies revealed greater advantages in those already taking corticosteroids to facilitate faster recovery and better clinical improvement than remdesivir alone antivirals are used in combination with the JAK inhibitors (JAK Inhibitors + Antivirals (e.g., Remdesivir (in ACTT-2 trial) to limit viral replication To combat the cytokine storm,.(JAK Inhibitors + IL-6 Inhibitors (e.g., Tocilizumab): both drugs are used, and both affect IL-6 signaling but through different mechanisms (Portsmore et al. 2020). Overlapping effects might not offer additive benefits and could increase infection risk. For that reason, clinical trials usually prefer one or the other, not both. JAK Inhibitors and Biologics: Although theoretically appealing, the combination of JAK inhibitors with biologic DMARDs, including anti-TNF or anti-IL-6 therapy, is usually avoided because of safety issues, especially opportunistic infections, and thromboembolic events. The majority of clinical trials exclude patients on the two therapies simultaneously, and regulatory recommendations are that these combinations are not to be employed. Off-label use in the context of refractory disease has been described in case series, and additional controlled studies are required to confirm safety and efficacy.

### Limitations of current Jak inhibitor drugs and possible alternate strategies to overcome them

Though Jak inhibitor drugs provide several advantages such as multi-target blocking and ease of administration over biologics, there are serious concerns related to the management and treatment of serious adverse effects such as gastrointestinal events, ulcerative colitis, serious infections, thrombotic complications, cardiovascular disorders, malignant tumors, and major risks of deaths, which are more significant than the use of biologics (Tanaka et al. 2022). The molecular basis of some of these includes overexpressed JAK3 downstream to MAPK signaling is reported to mediate platelet hyperactivation. Hence, neither Pan-Jak inhibitor nor JAK1-2 inhibitors are potent therapeutic options. Albeit clinical trials using Jak3 specific inhibitors are needed. In addition, data suggest use of irreversible Jak inhibitors is potentially dangerous due to the risk of severe immunodeficiency. Therefore, temporary use of competitive Jak inhibitors may provide relatively safer and efficacious alternative for treating the chronic inflammatory pathology including COVID-19 (Rommasi et al. 2022). Moreover, since JAK2 appears to be not involved in type I or type II interferon-signaling, selective Jak-2 specific inhibitors may provide a better outcome for suppressing IL-6-GM-CSF-signaling-in COVID-19-associated cytokine storms. Likewise, Jak inhibitors treatment also cause significant reduction in humoral immunity following vaccination against SARS-CoV-2 in rheumatoid arthritis patients. Therefore, future trails involving stratification of patients based of the stages of severity using blood cytokines to determine precise timing of the treatment may provide a better outcome. Since Jak inhibitors are an attractive alternate in combination with adjuvant therapy, more results are needed to establish the safety and efficacy particularly using suitable combination modalities under clinical setting. Moreover, the majority of results are based on relatively small sample size lacking head-to-head clinical trial comparisons. Therefore, data must be accumulated to establish drug safety with risk-free utilization of these inhibitors by specialists who can identify the potential patient cohorts who might benefit from these inhibitors the most. Though up to 2021, the FDA and EMA authorized seven Jak inhibitors for the treatment of various therapeutic conditions, in 2022 only, three new Jak inhibitors got approval from FDA for the first time, expanding the Jak inhibitor portfolio to a total of 10 approved Jak inhibitors for human and veterinary use, in United states. Since the discovery of Jaks over 40 years ago, the last three decades have witnessed Jak directed drug development undergo drastic translation from bench to bedside where they have become an integral part of therapeutic options for heterogenous group of ailments either as an alternative treatment for the conditions in which other biologics failed or as the sole mainstay treatment of conditions without prior approved therapeutic agents. The current hope is that with the use of better strategies these and many more Jak inhibitors will be able to address the unmet clinical needs of many more autoimmune and chronic inflammatory conditions including COVID-19.

### Prospective on oral *vs* trans mucosal delivery routes to administer Jak3 targeted drugs

Though a receptor for SARS-CoV-2, under physiological conditions, ACE2 protects against lung injury by: (a) degrading Ang II, which is vasoconstrictive and proapoptotic for lung epithelial cells and profibrotic, and (b) by producing the peptide Ang1-7, which inhibits the actions of Ang II through binding to the MAS receptor. In support of this protective role for ACE2, pharmaceutical preparations of recombinant ACE2, when administered to experimental animals, protect against lung cell death, inhibit acute lung injury and prevent lung fibrosis after chronic injury to the lungs (Samavati and Uhal 2020). In addition, MUC4 mucin, a ligand activates ErbB2, a receptor tyrosine kinase that modulates Airway Epithelial Cells (AEC) proliferation following damage in airways of asthmatic patients. The activation of Jak3 has been reported to upregulate the expression of both ACE2 and MUC4 in AEC (Lee et al. 2021; Damera et al. 2006). Jaks in general and Jak3 are widely expressed in both immune cells and intestinal epithelial cells (IECs) of both humans and mice. Structural implications of Jak3 domains beyond the immune cells and in gastrointestinal functions suggest that Jak functions are important for gastrointestinal wound repair and protection from predisposition to inflammatory bowel disease, obesity-associated metabolic syndrome, and epithelial cancers. In light of these, it is not surprising that Jak and Jak3 inhibitor treatment during COVID-19 leads to adverse effects including respiratory tract infection, gastrointestinal events, and ulcerative colitis (Table 1) and which appears to be because of interference with the protective roles of mucosal epithelial functions of these Jaks in airways and gastrointestinal tracts (Kumar et al. 2021). These suggest that Jaks have both beneficial functions through its actions in mucosal epithelial cells and detrimental functions during COVID-19 infections associated cytokine storm through uncontrolled activation of immune cells. Therefore, targeting immune cell functions during this infection may mitigate the adverse impacts and provide a better outcome through transmucosal drug administration as an alternative route.

**Table 1:** Completed, terminated, or undergoing clinical trials of some of the JAK inhibitors available around the world. JAK inhibitors are known for their severe adverse events (AE), such as respiratory failure, cardiac failure, venous thromboembolism, and severe infections, such as septic shock, pneumonia, herpes zoster-related diseases, malignancies, and more. Although some of these clinical trials were completed, only a few are currently approved in the United States for consumer use. Other products are either available in other countries, have been discontinued since it was found non-significant in their studies, or are in the process of determining safety and efficacy, like CPL409116. (References in the table are provided separately in the Supplemental material section)

ClinicalTrials.gov Identifier and Status	Drug	Route	Target	Primary Outcome	Phase	Adjuvant Therapy	Limitations/AE's	References
NCT03758443, Terminated	Izencitinib	Oral	Pan-JAK	1. Change from baseline in Total Mayo Score (tMS) at 8 weeks. 2. Phase 3 Maintenance: Number of participants who demonstrated clinical remission by adapted Mayo Score components at maintenance week (mWeek) 44.	Phase 3	N/A	The study was terminated early by company decision due to interim analysis results. Serious AEs: Colitis ulcerative.	(Alam et al. 2021)
NCT03732807, Completed	Ritlecitinib	Oral	JAK3/TEC	Percentage of participants with an absolute severity of alopecia tool (SALT) score of less than or equal to 20 at week 24 (time frame: week 24].	Phase 3	N/A	AEs: upper respiratory tract infection. Serious AEs: herpes zoster, serious infections.	(Parvathaneni and Gupta 2020)
NCT04401579, Completed	Baricitinib	Oral	JAK1/JAK2	Overall time to recovery from COVID-19, and time to recovery based on race, ethnicity, and sex.	Phase 3	Remdesivir	Serious AEs: respiratory failure	(Bellera et al. 2021)
NCT04404361, Terminated	Pacritinib	Oral	JAK2/IRAK1	Proportion of patients, infected with COVID-19, who progress to IMV and/or ECMO or death during the 28 days following randomization	Phase 3	Standard of Care	Serious AEs: cardiac events, bleeding, gastrointestinal events, infections (septic shock, pneumonia).	(Indari et al. 2021)
NCT04366232, Terminated	Ruxolitinib	Oral	JAK1/JAK2	At least 3 parameters in the biological criteria are met, including CRP and/or Ferritin among: (a) CRP: decrease > 50%, (b) Ferritinemia: decrease > 1/3, (c) Serum creatine: decrease > 1/3, (d) AST/ALT: decrease > 50%, (e) Eosinophils >50/mm3, (f) Lymphocytes > 1000/mm3	Phase 2	Standard of Care, Anakinra	The study was terminated by the investigator.	(Chakraborty et al. 2021)
NCT04359290, Completed	Ruxolitinib	Oral	JAK1/JAK2	Overall survival in subjects with severe COVID-19	Phase 2	Dexamethasone	Severe AEs: Life threatening pneumonia	(Branco et al. 2020)
NCT04377620, Completed	Ruxolitinib	Oral	JAK1/JAK2	Percentage of Participants Who Have Died Due to Any Cause	Phase 3	Standard of Care	Severe AEs: Abnormal laboratory parameters, renal/urinary disorders, respiratory disorders, vascular disorders.	(Shanker et al. 2024)
NCT04334044, Completed	Ruxolitinib	Oral	JAK1/JAK2	Recover of pneumonia characterized by cease of respiratory symptoms.	Phase 1 & 2	N/A	Severe AEs: abnormal laboratory parameters, hypertension, acute kidney injury, vascular device infection.	(Shanker et al. 2024)
NCT04402866, Completed	Nezulcitinib (TD-0903)	Oral Inhalation	Pan-JAK	Number of respiratory failure-free days from randomization to day 28.	Phase 2	Standard of Care, Corticosteroids, Heparin, Remdesivir	Severe AEs: acute respiratory failure, ventricular fibrillation, fatal MODS, and ischemic stroke.	(Satarker et al. 2021; Amini-Farsani et al. 2021)
NCT03281304, Terminated	Tofacitinib	Oral	JAK1/JAK2/JAK3	Number of participants, diagnosed with ulcerative colitis, with remission based on modified mayo score at month 6.	Phase 4	N/A	Severe AEs: herpes zoster, infections.	(McGee et al. 2021)
NCT02535689, Completed	Tofacitinib	Oral	JAK1/JAK2/JAK3	Safety of tofacitinib in systemic lupus erythematosus (SLE) patients.	Phase 1	Some eligible patients were on prednisone during the treatment period.	Severe AEs: Cardiac disorders, gastrointestinal disorders, infections, nervous system disorders.	(Zheng et al. 2021)
NCT02308163, Completed	Peficitinib	Oral	Pan-JAK	Percentage of Participants with an American College of Rheumatology 20% (ACR20) C-Reactive Protein (CRP) Response at Week 12	Phase 3	N/A	Severe AEs: serious infections, herpes zoster-related disease, malignances.	(Spinelli et al. 2020)
NCT01590459, Completed	Decernotinib	Oral	JAK3	Proportion of subjects who achieve a 20% improvement in disease severity according to the American College of Rheumatology criteria, assessed using the C-reactive protein level (ACR20-CRP) response, within twelve weeks.	Phase 2	Methotrexate	AEs: headaches, elevated levels of transaminases, lipoproteins, creatine, LDL and HDL cholesterol.	(Cantini et al. 2020)
NCT04670757, Completed	CPL409116	Oral	JAK1/JAK3/Pan-ROCK	(1) Determination of maximum tolerated dose (MTD) or administration of the maximum dose provided in the protocol after single and multiple oral administration of IMP, (2) Safety and tolerability of IMP after single and multiple oral administration	Phase 1	N/A	N/A	(Roschewski, et al. 2020; Jacobs et al. 2021)
NCT05374785, Recruiting	CPL409116	Oral	JAK1/JAK3/Pan-ROCK	Change in Disease Activity Score 28 joint count C reactive protein (DAS28(CRP)).	Phase 2	N/A	N/A	(Roschewski, et al. 2020; Jacobs et al. 2021)
NCT03728023, Completed	Golidocitinib (AZD4205)	Oral	JAK1	The number of healthy subjects with adverse events, abnormal laboratory parameters, abnormal vital signs, and abnormal electrocardiogram	Phase 1	N/A	AEs: Abnormal laboratory parameters (neutropenia, leukopenia, increased ALT, AST, and LDL), gastrointestinal and respiratory disorders.	(Basile et al. 2022; Roy et al. 2021)
NCT03450330, Completed	Golidocitinib (AZD4205)	Oral	JAK1	Safety and tolerability of AZD4205 in subjects with nonsmall cell lung cancer.	Phase 1 & 2	Osimertinib	AEs: Abnormal laboratory parameters (neutropenia, leukopenia, increased ALT, AST, and LDL), gastrointestinal and respiratory disorders.	(Basile et al. 2022; Roy et al. 2021)

With significant number of Jak inhibitors under clinical trials this combined with continued sustenance of COVID-19 pandemic deserves a need for alternative routes of administration of Jak inhibitor drugs for end of life and palliative care, particularly in community settings. Transmucosal routes include intranasal, buccal, sublingual, and rectal. In addition to being non-invasive routes for systemic drug delivery, it also provides the possibility of self-administration, or administration by family caregivers not to mention their ability to offer rapid onset of action with reduced first-pass metabolism making them suitable for use in palliative and end-of life care to provide fast relief of symptoms. This is particularly important in COVID-19, as patients can deteriorate rapidly. However, these alternate routes also come with challenges including a relatively small surface area for effective drug absorption, small volume of fluid for drug dissolution and the presence of a mucus barrier, that may limit the number of drugs that that may qualify for suitability for delivery through the transmucosal route (Lam et al. 2020).

## Concluding remarks

Janus Kinase 3 is a non-receptor tyrosine kinase belonging to the Jak family that is highly expressed in immune cells. Accumulating evidence including our research indicates that they are also expressed in a variety of non-immune cells tissues such as intestinal and lung epithelial cells (or mucosal jak3) and brain cells, liver cells. Mucosal jak3 are adjacent to the immune cells and immune cell Jak3 which is the reason behind the cytokine storm. So immune cell Jak3 is a potential target for covid treatment. Here we discuss the challenges and strategies to target these immune cell Jak3 with the Jak3 targeted drugs. As the understanding of the mucosal Jak3 and their intestinal tissue specific functions continue to improve, mucosal Jak3 and its role in maintaining the mucosal and immune cross talk will be identified. In that way the use of other formulation of Jak3 other than oral directed drugs will be explored to target immune cell Jak3. Immune cell Jak3 generally over activated in the covid patients leading to cytokine storm, which would allow the development of drugs acting at selective sites only. Advances in the basic knowledge of mucosal Jak3, their intestinal tissue specific functions, aboral formulation of Jak3 -directed drugs, and delivery systems will aid in the development of new therapeutic strategies targeting these immune cell Jak3 in combating covid while protecting the mucosal Jak3.

## Supplementary Information

Supplementary Material 1.

## Data Availability

No datasets were generated or analysed during the current study.
