# Correction: Mucosal implications of oral Jak3-targeted drugs in COVID patients

**DOI:** 10.1186/s10020-025-01359-3

**Published:** 2025-09-06

**Authors:** Narendra Kumar, Daniel Segovia, Priyam Kumar, Hima Bindu Atti, Soaham Kumar, Jayshree Mishra

**Affiliations:** 1 ILR-College of Pharmacy, Texas A&M University Health Science Center, Kingsville, TX USA; 2 University of Pennsylvania, Philadelphia, PA USA; 3 Veterans Memorial High School, Corpus Christi, TX USA

**Correction: Molecular Medicine 31, 203 (2025)**

**https://doi.org/10.1186/s10020-025-01260-z**

In the sentence beginning ‘More notably, the strong link between …. disease (Satarker et al. 2021).’ the term “SARS-CoV-2” is written as “SARS-Cov-2” in the section Tyrosine kinases in viral entry and replication of this article [1].

The corrected sentence should read as "More notably, the strong link between ACE2 & JAK-STAT signaling throughout the COVID-19 disease analysis was directly demonstrated by the above relationship involving ACE2 & IFN-JAK-STAT signaling being confirmed in a separate cell related to SARS-Cov and SARS-CoV-2 disease (Satarker et al. 2021)."

The original article has been corrected.
