# Supplementary material for: Mucosal implications of oral Jak3-targeted drugs in COVID patients
Source: Mol Med. 2025 May 23;31:203. doi: 10.1186/s10020-025-01260-z (PMC12100796; doi:10.1186/s10020-025-01260-z)
Supplement: Supplementary file 1 — Supplementary Material 1. [file 10020_2025_1260_MOESM1_ESM.pdf]

Supplemental materials

References included in the table 1

1. Sandborn WJ, Nguyen DD, Beattie DT, et al. Development of Gut-Selective Pan-Janus Kinase Inhibitor TD-1473 for Ulcerative Colitis: A Translational Medicine Programme. J Crohns Colitis. Sep 16 2020;14(9):1202-1213. doi:10.1093/ecco-jcc/jjaa049

2. Hardwick RN, Brassil P, Badagnani I, et al. Gut-Selective Design of Orally Administered Izencitinib (TD-1473) Limits Systemic Exposure and Effects of Janus Kinase Inhibition in Nonclinical Species. Toxicol Sci. Mar 28 2022;186(2):323-337. doi:10.1093/toxsci/kfac002

3. Kalil AC, Patterson TF, Mehta AK, et al. Baricitinib plus Remdesivir for Hospitalized Adults with Covid-19. N Engl J Med. Mar 4 2021;384(9):795-807. doi:10.1056/NEJMoa2031994

4. Cafardi J, Miller C, Terebelo H, et al. Efficacy and Safety of Pacritinib vs Placebo for Patients With Severe COVID-19: A Phase 2 Randomized Clinical Trial. JAMA Netw Open. Dec 1 2022;5(12):e2242918. doi:10.1001/jamanetworkopen.2022.42918

5. Eljaaly K, Malibary H, Alsulami S, Albanji M, Badawi M, Al-Tawfiq JA. Description and Analysis of Cytokine Storm in Reg-istered COVID-19 Clinical Trials: A Systematic Review. Pathogens. Jun 2 2021;10(6)doi:10.3390/pathogens10060692

1. Sandborn WJ, Nguyen DD, Beattie DT, et al. Development of Gut-Selective Pan-Janus Kinase Inhibitor TD-1473 for Ulcerative Colitis: A Translational Medicine Programme. J Crohns Colitis. Sep 16 2020;14(9):1202-1213. doi:10.1093/ecco-jcc/jjaa049

2. Hardwick RN, Brassil P, Badagnani I, et al. Gut-Selective Design of Orally Administered Izencitinib (TD-1473) Limits Systemic Exposure and Effects of Janus Kinase Inhibition in Nonclinical Species. Toxicol Sci. Mar 28 2022;186(2):323-337. doi:10.1093/toxsci/kfac002

3. Kalil AC, Patterson TF, Mehta AK, et al. Baricitinib plus Remdesivir for Hospitalized Adults with Covid-19. N Engl J Med. Mar 4 2021;384(9):795-807. doi:10.1056/NEJMoa2031994

4. Cafardi J, Miller C, Terebelo H, et al. Efficacy and Safety of Pacritinib vs Placebo for Patients With Severe COVID-19: A Phase 2 Randomized Clinical Trial. JAMA Netw Open. Dec 1 2022;5(12):e2242918. doi:10.1001/jamanetworkopen.2022.42918

5. Eljaaly K, Malibary H, Alsulami S, Albanji M, Badawi M, Al-Tawfiq JA. Description and Analysis of Cytokine Storm in Reg-istered COVID-19 Clinical Trials: A Systematic Review. Pathogens. Jun 2 2021;10(6)doi:10.3390/pathogens10060692

6. Neubauer A, Johow J, Mack E, et al. The janus-kinase inhibitor ruxolitinib in SARS-CoV-2 induced acute respiratory distress syndrome (ARDS). Leukemia. Oct 2021;35(10):2917-2923. doi:10.1038/s41375-021-01374-3

7. Rein L, Calero K, Shah R, et al. Randomized Phase 3 Trial of Ruxolitinib for COVID-19-Associated Acute Respiratory Distress Syndrome. Crit Care Med. Dec 1 2022;50(12):1701-1713. doi:10.1097/CCM.0000000000005682

8. Singh D, Bogus M, Moskalenko V, et al. A phase 2 multiple ascending dose study of the inhaled pan-JAK inhibitor nezulcitinib (TD-0903) in severe COVID-19. Eur Respir J. Oct 2021;58(4)doi:10.1183/13993003.00673-2021

9. Pfeifer ND, Lo A, Bourdet DL, Colley K, Singh D. Phase I study in healthy participants to evaluate safety, tolerability, and pharmacokinetics of inhaled nezulcitinib, a potential treatment for COVID-19. Clin Transl Sci. Nov 2021;14(6):2556-2565. doi:10.1111/cts.13123

10. Winthrop KL, Vermeire S, Long MD, et al. Long-term Risk of Herpes Zoster Infection in Patients With Ulcerative Colitis Receiving Tofacitinib. Inflamm Bowel Dis. Jan 5, 2023;29(1):85-96. doi:10.1093/ibd/izac063

11. Hasni SA, Gupta S, Davis M, et al. Phase 1 double-blind randomized safety trial of the Janus kinase inhibitor tofacitinib in systemic lupus erythematosus. Nat Commun. Jun 7, 2021;12(1):3391. doi:10.1038/s41467-021-23361-z

12. Tanaka Y, Takeuchi T, Tanaka S, et al. Efficacy and safety of peficitinib (ASP015K) in patients with rheumatoid arthritis and an inadequate response to conventional DMARDs: a randomised, double-blind, placebo-controlled phase III trial (RAJ3). Ann Rheum Dis. Oct 2019;78(10):1320-1332. doi:10.1136/annrheumdis-2019-215163

13. Genovese MC, van Vollenhoven RF, Pacheco-Tena C, Zhang Y, Kinnman N. VX-509 (Decernotinib), an Oral Selective JAK-3 Inhibitor, in Combination with Methotrexate in Patients With Rheumatoid Arthritis. Arthritis Rheumatol. Jan 2016;68(1):46-55. doi:10.1002/art.39473

14. Dulak-Lis M, Bujak A, Gala K, et al. A novel JAK/ROCK inhibitor, CPL409116, demonstrates potent efficacy in the mouse model of systemic lupus erythematosus. J Pharmacol Sci. Apr 2021;145(4):340-348. doi: 10.1016/j.jphs.2021.02.002

15. Gurba-Bryskiewicz L, Dawid U, Smuga DA, et al. Implementation of QbD Approach to the Development of Chromatographic Methods for the Determination of Complete Impurity Profile of Substance on the Preclinical and Clinical Step of Drug Discovery Studies. Int J Mol Sci. Sep 14 2022;23(18) doi:10.3390/ijms231810720

16. Chen K, Guan X, Yang Z, et al. Pharmacokinetic characteristics of golidocitinib, a highly selective JAK1 inhibitor, in healthy adult participants. Front Immunol. 2023; 14:1127935. doi:10.3389/fimmu.2023.1127935

17. Su Q, Banks E, Bebernitz G, et al. Discovery of (2R)-N-[3-[2-[(3-Methoxy-1-methyl-pyrazol-4-yl)amino]pyrimidin-4-yl]-1H-indol-7-yl]-2-(4-methylpiperazin-1-yl)propenamide (AZD4205) as a Potent and Selective Janus Kinase 1 Inhibitor. J Med Chem. May 14 2020;63(9):4517-4527. doi:10.1021/acs.jmedchem.9b01392
